# Supplementary material for: Inverse relationship between species competitiveness and intraspecific trait variability may enable species coexistence in experimental seedling communities
Source: Nat Commun. 2024 Apr 3;15:2895. doi: 10.1038/s41467-024-47295-4 (PMC10991546; doi:10.1038/s41467-024-47295-4)
Supplement: Supplementary file 1 — Supplementary Information [file 41467_2024_47295_MOESM1_ESM.docx]

**Supplementary Information** for

**Inverse relationship between species competitiveness and intraspecific trait variability may enable species coexistence in experimental seedling communities**

Jing Yang^1^, Xiya Wang^1^, Carlos P. Carmona^2^, Xihua Wang^1,3^, Guochun Shen^1,3^*

*^1^Zhejiang Tiantong Forest Ecosystem National Observation and Research Station, School of Ecological and Environmental Science, East China Normal University, Shanghai, 200241 China*

*^2^Institute of Ecology and Earth Sciences, University of Tartu, Tartu, Estonia*

*^3^Shanghai Institute of Pollution Control and Ecological Security, 1515 North Zhongshan Rd. (No.2), Shanghai, 200092 China*

**This file includes:**

**Table S1 to S10**

**Fig. S1 to S15**

**Supplementary Tables**

**Table S1** Summary of the mixed-effects models employed to explore the relationships involving competitive treatments (competition-free and competition, abbreviated as ‘CompTreat’), competitiveness (*RII*), and their interaction concerning species’ ITV within the context of multispecies experiment. These analyses were separately conducted in both homogeneous (specifically, environmental block 1, which corresponds to the Phase I environment) and heterogeneous environments, with species and their average intial height as a random factor. Significance of fixed effects was tested by two-sided Student’s *t*-test.

|  | | Predictors | Estimates | CI | *P*-value |
| --- | --- | --- | --- | --- | --- |
| Multispecies experiment in a homogeneous environment | CompTreat_competition-free | | -0.41 | -2.20 – 3.68 | 0.570 |
|  | CompTreat_competition | | -0.51 | -2.60 – 3.27 | 0.796 |
|  | competitiveness | | -0.01 | -5.65 – 4.24 | 0.746 |
|  | CompTreat_competition* competitiveness | | -7.14 | -13.97 – -1.32 | 0.043 |
|  | | | | Pseudo *R^2^* | 0.871 |
| Multispecies experiment in a heterogeneous environment | CompTreat_competition-free | | -0.10 | -3.43 – 3.34 | 0.948 |
|  | CompTreat_competition | | 0.32 | -3.01 – 3.66 | 0.825 |
|  | competitiveness | | -8.70 | -14.56 – -2.84 | 0.010 |
|  | CompTreat_competition* competitiveness | | -6.26 | -12.13 – -0.39 | 0.040 |
|  | | | | Pseudo *R^2^* | 0.900 |

**Table S2** The relative competitive intensity index (*RII*, mean ± standard error) of competing species from all seedlings in the paired or mixed cultures in our two-phase experiment. The former value in the sample sizes represents the count of surviving seedlings at harvest, utilized for *RII* calculation. The latter value denotes the sample size used for ITV calculation after the removal of individuals with missing traits. *V* and *P* (asterisk in Table 2) values are derived from the two-sided Wilcoxon rank sum test. P<0.05 indicate that the species, compared to the competition-free treatment, are subjected to a significant biomass suppression due to competition.

| Phase | Pair code | Species | *RII* | sample size under competition-free | sample size under competition | *V* | *P*-value |
| --- | --- | --- | --- | --- | --- | --- | --- |
| Phase I | CM-QC | CM | -0.36±0.02 | 43/44 | 53/49 | 0 | 2.46e-10 |
|  |  | QC | -0.18±0.02 | 45/45 | 53/52 | 96 | 4.25e-08 |
|  | CM-CG | CM | -0.25±0.03 | 46/44 | 43/41 | 12 | 2.69e-08 |
|  |  | CG | -0.2±0.02 | 43/43 | 43/39 | 14 | 3.08e-08 |
|  | CS-CG | CS | -0.24±0.04 | 45/43 | 44/40 | 80 | 9.07e-08 |
|  |  | CG | -0.23±0.03 | 43/43 | 44/44 | 64 | 5.05e-07 |
|  | PS-HA | PS | -0.38±0.02 | 45/42 | 45/42 | 4 | 7.01e-09 |
|  |  | HA | -0.09±0.02 | 45/44 | 45/45 | 194 | 2.66e-04 |
|  | QC-HA | QC | -0.31±0.04 | 45/45 | 43/42 | 28 | 7.99e-08 |
|  |  | HA | -0.11±0.03 | 45/44 | 43/41 | 191 | 6.75e-04 |
|  | SS-LH | SS | -0.4±0.04 | 44/42 | 44/36 | 3 | 5.58e-13 |
|  |  | LH | -0.22±0.03 | 45/41 | 44/44 | 73 | 8.70e-07 |
|  | PS-QC | PS | -0.42±0.02 | 45/42 | 44/43 | 0 | 7.87e-09 |
|  |  | QC | -0.16±0.03 | 45/45 | 44/43 | 79 | 8.24e-08 |
|  | CG-QC | CG | -0.36±0.04 | 43/43 | 33/31 | 1 | 4.66e-10 |
|  |  | QC | 0.002±0.03 | 45/45 | 33/27 | 328 | 0.401 |
| Phase II | Homogeneous environment | | | | | | |
|  | — | DO | -0.77±0.02 | 19/19 | 18/17 | 0 | 7.63e-06 |
|  | — | LG | -0.70±0.03 | 20/20 | 19/18 | 0 | 3.82e-06 |
|  | — | CS | -0.68±0.03 | 20/20 | 20/18 | 0 | 1.91e-06 |
|  | — | CG | -0.61±0.03 | 20/20 | 20/20 | 0 | 1.91e-06 |
|  | — | HA | -0.52±0.05 | 17/17 | 20/18 | 1 | 3.82e-06 |
|  | — | SS | -0.40±0.04 | 16/16 | 18/15 | 0 | 7.63e-06 |
|  | — | QC | -0.35±0.04 | 20/20 | 20/15 | 0 | 1.91e-06 |
|  | Heterogeneous environment | | | | | | |
|  | — | DO | -0.73±0.02 | 142/132 | 137/120 | 1 | <2.2e-16 |
|  | — | LG | -0.64±0.02 | 172/170 | 177/147 | 0 | <2.2e-16 |
|  | — | CS | -0.63±0.01 | 163/123 | 163/114 | 0 | <2.2e-16 |
|  | — | CG | -0.58±0.02 | 174/174 | 176/165 | 0 | <2.2e-16 |
|  | — | HA | -0.43±0.02 | 151/122 | 170/114 | 594 | <2.2e-16 |
|  | — | SS | -0.56±0.02 | 156/128 | 150/99 | 6 | <2.2e-16 |
|  | — | QC | -0.30±0.02 | 164/156 | 175/157 | 394 | <2.2e-16 |

**Table S3** Means and standard errors of changes in intraspecific variability for competitively inferior and competitively superior per species pair in Phase I. For all species pairs, *V* and *P* values are derived from the Wilcoxon Signed-rank sum test based on species level. For each species pair, *V* and *P* values are derived from the one-sided Wilcoxon rank sum test based 999 replications changes of hypervolume.

| Pair code | Changes of intraspecific variability | | *V* | *P*-value |
| --- | --- | --- | --- | --- |
|  | inferiors | superiors |  |  |
| All pairs | 30.67±0.52 | -0.5±0.13 | 35 | 0.007 |
| CM-QC | 6.34±0.27 | 0.27±0.01 | 741582 | 5.83e-79 |
| CM-CG | 7.23±0.26 | -5.98±0.17 | 927256 | 6.76e-242 |
| CS-CG | 56.95±1.8 | 4.24±0.35 | 821077 | 1.03e-137 |
| PS-HA | -0.85±0.06 | -0.38±0.02 | 357927 | 7.33e-28 |
| QC-HA | 2.84±0.04 | -0.1±0.02 | 993717 | 0 |
| SS-LH | 106.08±1.81 | -4.45±0.91 | 972950 | 8.70e-296 |
| PS-QC | 18.03±0.36 | 0.85±0.02 | 984743 | 0 |
| CG-QC | 48.77±0.98 | 1.53±0.01 | 971544 | 4.78e-294 |

**Table S4** Summary of coefficient estimates of models testing the relationship between *RII* and changes of intraspecific variability from competition-free to competition (Figure 3) environments. Significance of fixed effects was tested by two-sided Student’s *t*-test.

|  | Slope | S. E. | *t*-value | *N* | *P*-value | Adjusted *R^2^* |
| --- | --- | --- | --- | --- | --- | --- |
| Total | -13.25 | 1.95 | -6.81 | 30 | 2.14e-07 | 0.61 |
| homogeneous environment | -9 | 2.83 | -3.18 | 7 | 0.025 | 0.60 |
| heterogeneous environment | -13.66 | 2.39 | -5.72 | 7 | 0.002 | 0.84 |

**Table S5** Means and standard errors of changes in intraspecific trait variability for each functional trait from competition-free to competition. Positive and negative values represent an increase and decrease in the relative variability of a target trait under competition, respectively. *V* and *P* values are derived from the one-sided Wilcoxon rank sum test for competing species.

| Phase | Trait | Mean changes of ITV | N | *V* | *P*-value |
| --- | --- | --- | --- | --- | --- |
| Total | Chl | 0.06 ± 0.01 | 30 | 428 | 0 |
|  | LMA | 0.01 ± 0.01 | 30 | 80 | 0.562 |
|  | LDMC | 0.03 ± 0.01 | 30 | 376 | 0.002 |
|  | LTO | -0.01 ± 0.01 | 30 | 203 | 0.556 |
|  | LTh | 0 ± 0.01 | 30 | 66 | 0.94 |
|  | SSD | 0.04 ± 0.01 | 30 | 380 | 0.002 |
|  | SMC | 0.06 ± 0.02 | 30 | 115 | 0.013 |
|  | SRL | 0.23 ± 0.04 | 30 | 104 | 0 |
|  | SRA | 0.16 ± 0.03 | 30 | 105 | 0 |
|  | RTD | 0.27 ± 0.04 | 30 | 105 | 0 |
| Pairwise competition in a homogeneous environment | Chl | 0.06 ± 0.02 | 16 | 129 | 0.001 |
|  | LMA | 0.01 ± 0.01 | 16 | 80 | 0.561 |
|  | LDMC | 0.02 ± 0.01 | 16 | 102 | 0.083 |
|  | LTO | 0 ± 0.01 | 16 | 65 | 0.900 |
|  | LTh | 0 ± 0.01 | 16 | 66 | 0.940 |
|  | SSD | 0.04 ± 0.02 | 16 | 102 | 0.083 |
|  | SMC | 0.06 ± 0.02 | 16 | 115 | 0.013 |
| Multispecies competition in a homogeneous environment | Chl | 0.07 ± 0.03 | 7 | 25 | 0.078 |
|  | LDMC | 0.03 ± 0.01 | 7 | 25 | 0.078 |
|  | LTO | 0.02 ± 0.02 | 7 | 19 | 0.469 |
|  | SSD | 0.03 ± 0.01 | 7 | 25 | 0.078 |
|  | SRL | 0.36 ± 0.04 | 7 | 28 | 0.016 |
|  | SRA | 0.24 ± 0.04 | 7 | 28 | 0.016 |
|  | RTD | 0.34 ± 0.06 | 7 | 28 | 0.016 |
| Multispecies competition in a heterogeneous environment | Chl | 0.07 ± 0.03 | 7 | 28 | 0.016 |
|  | LDMC | 0.05 ± 0.03 | 7 | 25 | 0.078 |
|  | LTO | -0.04 ± 0.02 | 7 | 4 | 0.109 |
|  | SSD | 0.04 ± 0.02 | 7 | 26 | 0.047 |
|  | SRL | 0.1 ± 0.02 | 7 | 27 | 0.031 |
|  | SRA | 0.08 ± 0.02 | 7 | 28 | 0.016 |
|  | RTD | 0.19 ± 0.02 | 7 | 28 | 0.016 |

**Table S6** Mean changes in interspecific trait dissimilarity from competition-free to competitive treatments for 8 paired species in Phase I. Interspecific trait dissimilarity was quantified by the centroid distance of the hypervolume between paired species (sample size of 20 per species pair, replicated 999 times). Positive and negative values represent an increase and decrease in interspecific trait dissimilarity under competition, respectively. The statistical significance (*P*) of these changes was assessed using two-sided Wilcoxon rank sum tests for competing species.

| species | Mean changes of interspecific trait dissimilarity | *V* | *P*-value |
| --- | --- | --- | --- |
| All pairs | 0.057±0.003 | 19929782 | < 0.001 |
| CM-QC | -0.239±0.004 | 975 | < 0.001 |
| CM-CG | -0.025±0.005 | 202650 | < 0.001 |
| CS-CG | -0.166±0.005 | 32753 | < 0.001 |
| PS-HA | 0.029±0.006 | 286313 | < 0.001 |
| QC-HA | 0.06±0.005 | 345546 | < 0.001 |
| SS-LH | 0.213±0.006 | 468451 | < 0.001 |
| PS-QC | 0.301±0.004 | 499254 | < 0.001 |
| CG-QC | 0.283±0.004 | 498783 | < 0.001 |

**Table S7** General information of the tree species used in our two-phase seedling experiment.

| Phase | Latin name | Abbr. ^†^ | Genus | Family | Growth form | Number of seedlings | | Height of transplanted seedlings (cm) |
| --- | --- | --- | --- | --- | --- | --- | --- | --- |
| Phase I/II | *Quercus chenii* | QC | Quercus | Fagaceae | DC^‡^ | 700/1600 | 18.0 – 25.1 | |
| Phase I/II | *Hovenia acerba* | HA | Hovenia | Rhamnaceae | DC | 450/1300 | 12.5 – 17.2 | |
| Phase I/II | *Castanopsis sclerophylla* | CS | Castanopsis | Fagaceae | EG | 300/1100 | 6.2 – 9.8 | |
| Phase I/II | *Schima superba* | SS | Schima | Theaceae | EG | 300/1100 | 6.1 – 8.3 | |
| Phase I/II | *Cyclobalanopsis glauca* | CG | Cyclobalanopsis | Fagaceae | EG | 600/1400 | 8.1 – 12.3 | |
| Phase I | *Lithocarpus harlandii* | LH | Lithocarpus | Fagaceae | EG | 300 | 8.0 – 11.2 | |
| Phase I | *Cyclobalanopsis myrsinifolia* | CM | Cyclobalanopsis | Fagaceae | EG | 500 | 8.7 – 12.3 | |
| Phase I | *Phoebe sheareri* | PS | Phoebe | Lauraceae | EG | 500 | 8.9 – 12.7 | |
| Phase II | *Daphniphyllum oldhami* | DO | Daphniphyllum | Daphniphyllaceae | EG | 1100 | 7.0 – 9.7 | |
| Phase II | *Lithocarpus glaber* | LG | Lithocarpus | Fagaceae | EG | 1300 | 7.1 – 10.0 | |
| Phase II | *Phoebe chekiangensis* | PC | Phoebe | Lauraceae | EG | 500 | 7.6 – 9.0 | |
| Phase II | *Aphananthe aspera* | AA | Aphananthe | Ulmaceae | DC | 200 | 13.5 – 18.9 | |
| Phase II | *Celtis sinensis* | CS | Celtis | Ulmaceae | DC | 200 | 16.1 – 23.4 | |
| Phase II | *Mallotus japonicus* | MJ | Mallotus | Euphorbiaceae | DC | 400 | 15.3 – 21.0 | |
| Phase II | *Elaeocarpus decipiens* | ED | Elaeocarpus | Elaeocarpaceae | EG | 500 | 9.1 – 14.5 | |
| Phase II | *Ilex micrococca* | IM | Ilex | Aquifoliaceae | EG | 500 | 6.3 – 8.5 | |

Note: Phase I: the pairwise competition experiment in a homogeneous environment (Figure 1a); Phase II: the multispecies competition experiment in the homogeneous (Figure 1b) and heterogeneous (Figure 1c) environment; Number of seedlings: the number of seedlings germinating successfully from seed and germination in Phase I/II corresponds to the front and back of the slash, respectively. †: Abbreviation of species name; ‡: DC: deciduous; EG: evergreen. The height of transplanted seedlings was the interquartile range of the height of all planted seedling.

**Table S8** Light intensity, soil moisture and soil phosphorus (total and effective phosphorus) in nine environmental blocks during Phase II.

| Environment block | Light (μmol/(m^2^⋅s)) | Soil Moisture  (%) | Soil phosphorus | |
| --- | --- | --- | --- | --- |
|  |  |  | TP (g/kg) | AP (mg/kg) |
| Block 1 | High  1237.20 ± 195.45 | High  33.00±0.36 | Low  0.66±0.07 | Low  139.03±34.81 |
| Block 2 | High  1237.20 ± 195.45 | Low  17.91±0.25 | High  1.1±0.06 | High  364.55±10.89 |
| Block 3 | High  1237.20 ± 195.45 | Medium  29.91±0.24 | Medium  0.92±0.02 | Medium  154.12±6.23 |
| Block 4 | Low  14.54 ± 1.99 | Medium  27.83±0.16 | Low  0.33±0.00 | Low  2.98±0.33 |
| Block 5 | Low  14.54 ± 1.99 | Low  26.81±0.39 | Medium  0.95±0.02 | Medium  255.13±13.75 |
| Block 6 | Low  14.54 ± 1.99 | High  31.92±0.31 | High  0.98±0.04 | High  267.03±12.23 |
| Block 7 | Medium  502.91 ± 103.09 | High  34.20±0.16 | Medium  0.80±0.01 | Medium  172.42±6.07 |
| Block 8 | Medium  502.91 ± 103.09 | Low  19.35±0.21 | Low  0.49±0.01 | Low  24.11±1.97 |
| Block 9 | Medium  502.91 ± 103.09 | Medium  28.55±0.27 | High  1.15±0.04 | High  253.08±20.60 |

Note: TP: Total phosphorus; AP: Available phosphorous; The light intensity was quantified as the maximum value and standard error of the light intensity during a summer day from 8:00 AM to 5:00 PM.

**Table S9** List of functional traits measured in this study.

| Plant organs | Trait name | Abbreviations | Units | Phase |
| --- | --- | --- | --- | --- |
| leaf | Chlorophyll content | Chl | SPAD | Phase I/II |
|  | leaf mass per area | LMA | g/cm^2^ | Phase I |
|  | leaf dry matter content | LDMC | mg/g | Phase I/II |
|  | leaf toughness | LTO | GN | Phase I/II |
|  | leaf thickness | LTh | mm | Phase I |
| stem | stem specific density | SSD | g/cm^3^ | Phase I/II |
|  | stem moisture content | SMC | g/g | Phase I |
| root | specific root length | SRL | cm/g | Phase II |
|  | specific root area | SRA | cm^2^/g | Phase II |
|  | root tissue density | RTD | g/cm^3^ | Phase II |

**Table S10** Trait scores and trait explanatory power of the different principal component axes in the results of principal component analysis of species trait data in two-phase experiments.

| Phase | Trait | PC1 | PC2 | PC3 | PC4 | PC5 | PC6 | PC7 |
| --- | --- | --- | --- | --- | --- | --- | --- | --- |
| Phase I | LTh | 0.48 | 0.02 | 0.25 | 0.27 | 0.01 | 0.45 | 0.65 |
|  | LDMC | 0.21 | 0.41 | -0.84 | -0.01 | 0.15 | -0.09 | 0.22 |
|  | LMA | 0.49 | 0.16 | -0.03 | 0.16 | 0.06 | 0.42 | -0.72 |
|  | Chl | 0.4 | 0.16 | 0.2 | -0.87 | -0.01 | -0.09 | 0.05 |
|  | LTO | 0.47 | 0.08 | 0.2 | 0.36 | -0.13 | -0.76 | -0.04 |
|  | WD | -0.22 | 0.65 | 0.13 | 0.04 | -0.71 | 0.11 | 0.01 |
|  | WMC | 0.23 | -0.6 | -0.36 | -0.09 | -0.67 | 0.06 | 0 |
|  | Variation (%) | 53.9 | 24.49 | 10.97 | 6.01 | 2.69 | 1.35 | 0.58 |
| Phase II | Chl | 0.25 | 0.54 | 0.1 | 0.12 | 0.77 | 0.17 | 0.02 |
|  | LTO | 0.33 | 0.12 | -0.25 | 0.83 | -0.32 | 0.14 | -0.06 |
|  | LDMC | 0.45 | -0.28 | -0.43 | -0.05 | 0.27 | -0.67 | -0.06 |
|  | SSD | 0.33 | -0.52 | -0.27 | -0.19 | 0.17 | 0.7 | 0.04 |
|  | SRL | -0.38 | 0.23 | -0.67 | -0.03 | 0.03 | 0.04 | 0.6 |
|  | RTD | 0.57 | 0.17 | 0.3 | -0.23 | -0.32 | -0.05 | 0.64 |
|  | SRA | 0.22 | 0.51 | -0.36 | -0.45 | -0.33 | 0.11 | -0.48 |
|  | Variation (%) | 30.04 | 24.83 | 17.83 | 12.58 | 7.70 | 4.88 | 0.21 |

**Supplementary Figures**

**
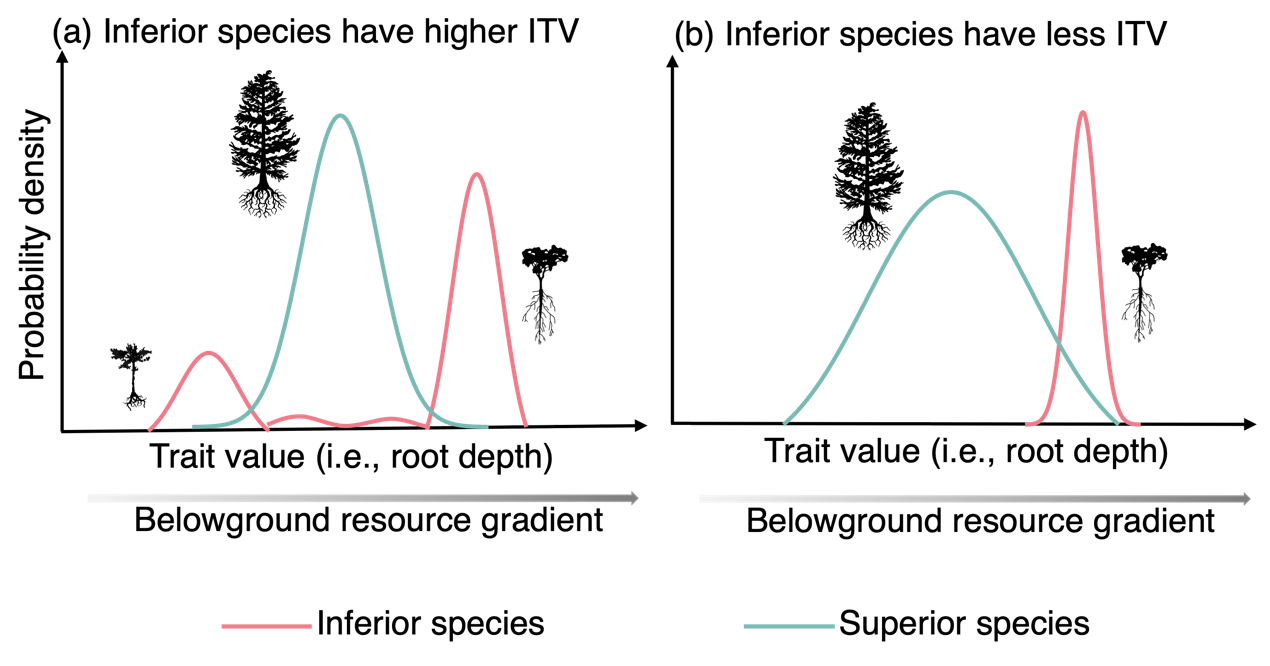
**

**Fig. S1** **|** Two distinct scenarios describe the relative magnitude of intraspecific trait variability (ITV) in competitively superior (represented by a green curve) and competitively inferior species (represented by a red curve). Scenario (a) depicts the inferior competitor having greater ITV than superior competitor. Conversely, scenario (b) suggests that inferior species have less ITV than superior competitor. Using root depth (as a proxy for underground resource utilization) as an example, superior competitor occupy a few underground resource niches, while the inferior species, either through active adaptation or passive exploitation, inhabit the remaining accessible niches. This leads to a non-continuous, multimodal trait distribution, culminating in a heightened ITV for root depth. In contrast, scenario (b) presents inferior species as having less ITV relative to superior competitor, possibly due to their inherent limitations in trait plasticity or adaptability, which may contribute to their competitive disadvantage. The red solid lines alongside the green solid line depict the trait distributions for inferior and superior competitor, respectively. The visual elements used in this figure are provided by the Integration and Application Network (IAN, ian.umces.edu) at the University of Maryland Center for Environmental Science (UMCES), which are available under the Creative Commons Attribution-ShareAlike 4.0 International (CC BY-SA 4.0) license (https://creativecommons.org/licenses/by-sa/4.0/).


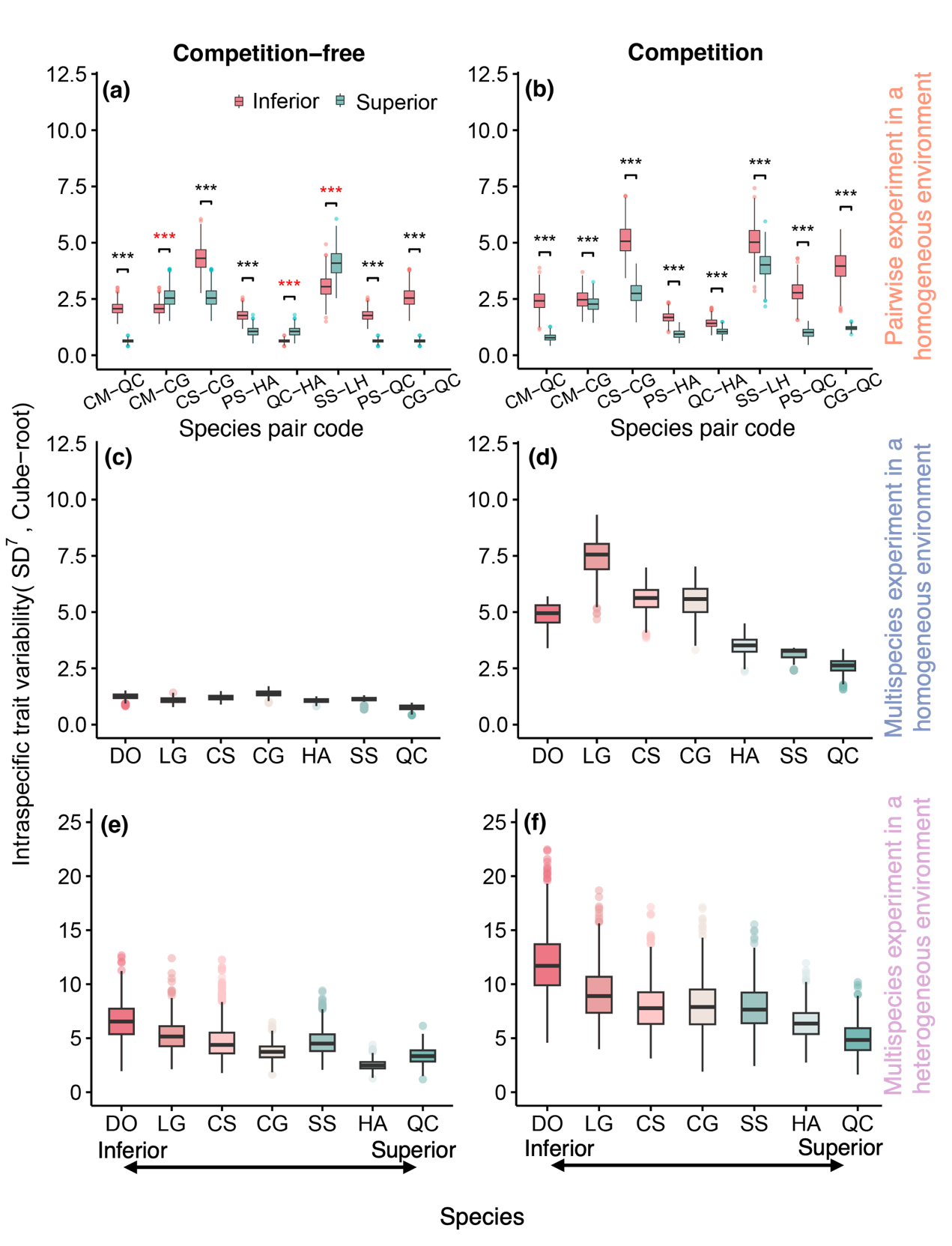


**Fig. S2 |** Intraspecific trait variability (cube-root transformation, quantified by the hypervolume size based on 7 key traits) for competitively inferior and competitively superior per species pair in a 2-species pairwise competition experiment in a homogeneous environment (a-b), per species in multispecies experiment in homogeneous (c-d, experimental block 1) and heterogeneous (e-f) environments. The left and right panels are for competition-free and competition treatments, respectively. Black asterisks (a-b) between the boxes suggest that inferior competitor had significantly larger hypervolume sizes than superior competitor, while red asterisks indicate inferior species had significantly smaller hypervolume sizes than superior competitor (•:<0.1; *: <0.05; **: <0.01; ***: <0.001). Intraspecific trait variability was quantified by multidimensional hypervolume size (SD^7^ units) constructed from same (20 in Phase I, 15 in Phase II) randomly selected samples and replicated 999 times. Detailed species Latin names for each species pair code are given in Table 1.


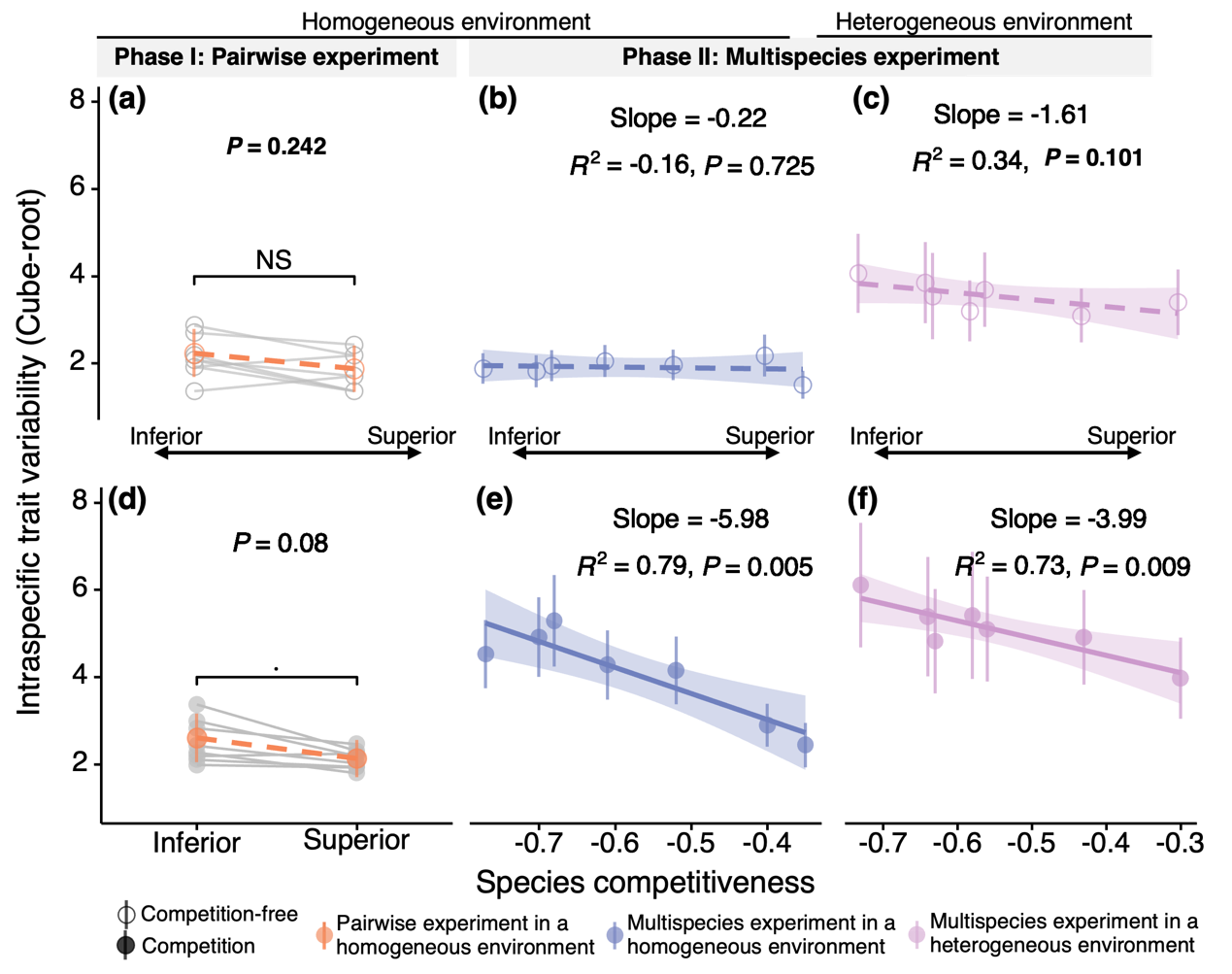


**Fig. S3** | Relationships between reduced dimensional intraspecific trait variability (ITV) and competitiveness of species. a-c, marked with hollow points, denote competition-free treatments, whereas d-f, marked with solid points, represent three varied competition treatments. Specifically, a, d depicts pairwise experiments in homogeneous environments (n = 345 and 661 seedlings, respectively), b, e and c, f depict multispecies experiments in homogeneous (n = 132 and 121 seedlings, respectively) and heterogeneous (n = 1005 and 916 seedlings, respectively) environments, respectively. Intraspecific trait variability was quantified by the reduced dimensional (the first 3 principal axes) hypervolume, measured in SD^3^ units. Competitiveness was quantified using the relative interaction intensity index (*RII*) based on whole seedlings’ biomass. Gray (a, d), blue (b, e), and purple (c, f) points with error bars represent the mean and standard error of the 999 simulated hypervolumes for each competing species, transformed by cube root. Detailed sample sizes for each competing species in two-phase experiment are shown in Table S4. The orange dots in a and d represent the mean ITVs of the eight superior (or inferior) species. The gray and orange line connecting these dots visually depicts the difference in ITV between competing species, and significance was tested by one-sided paired Wilcoxon rank-sum test. Black asterisks indicate levels of significance (•: <0.1; *: <0.05; **: <0.01; ***: <0.001). NS indicates nonsignificant. In b, c and d–f, solid (*P* < 0.05) and dashed (*P* > 0.05) lines are simple linear regression lines with 95% confidence intervals.


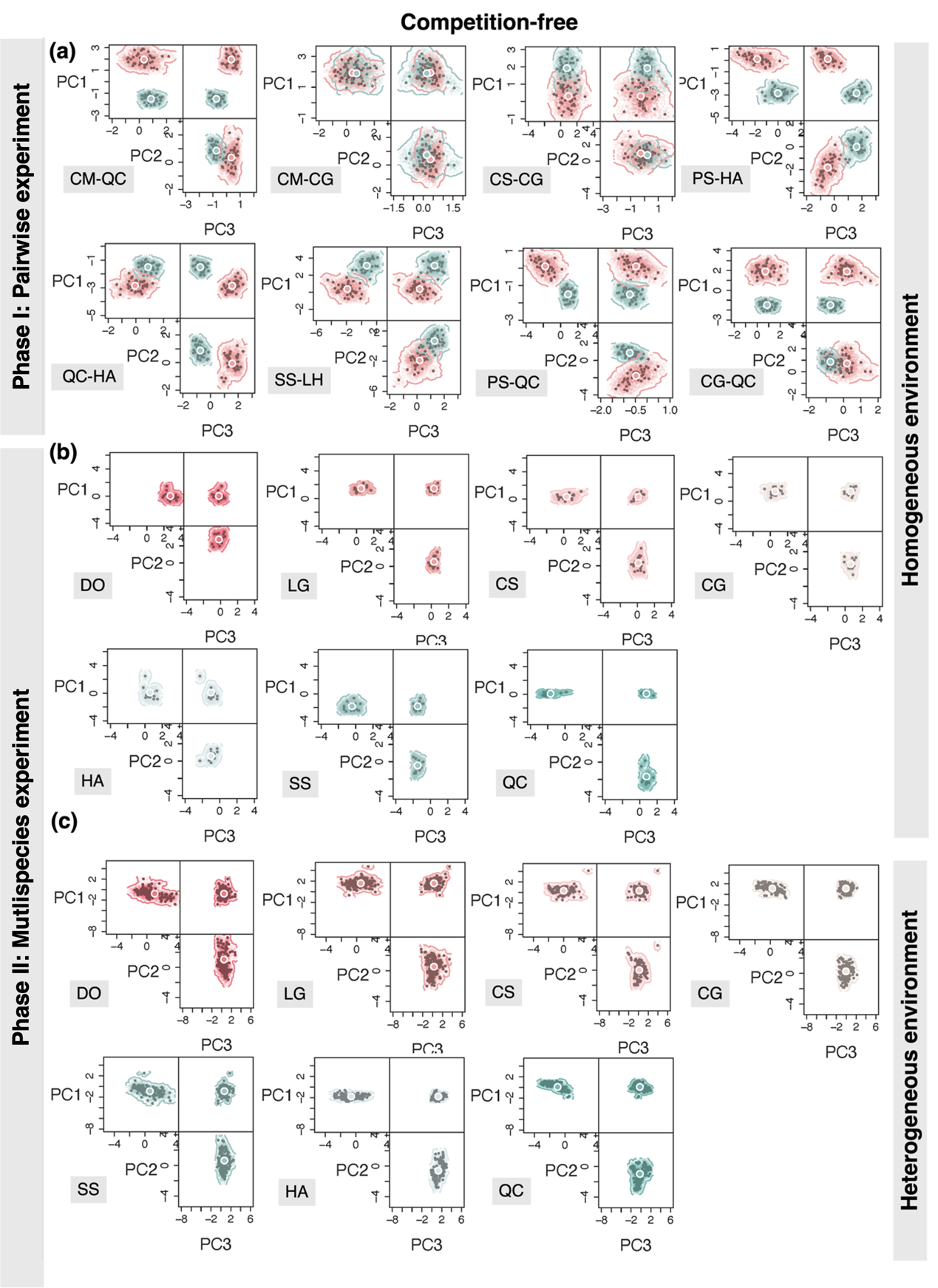


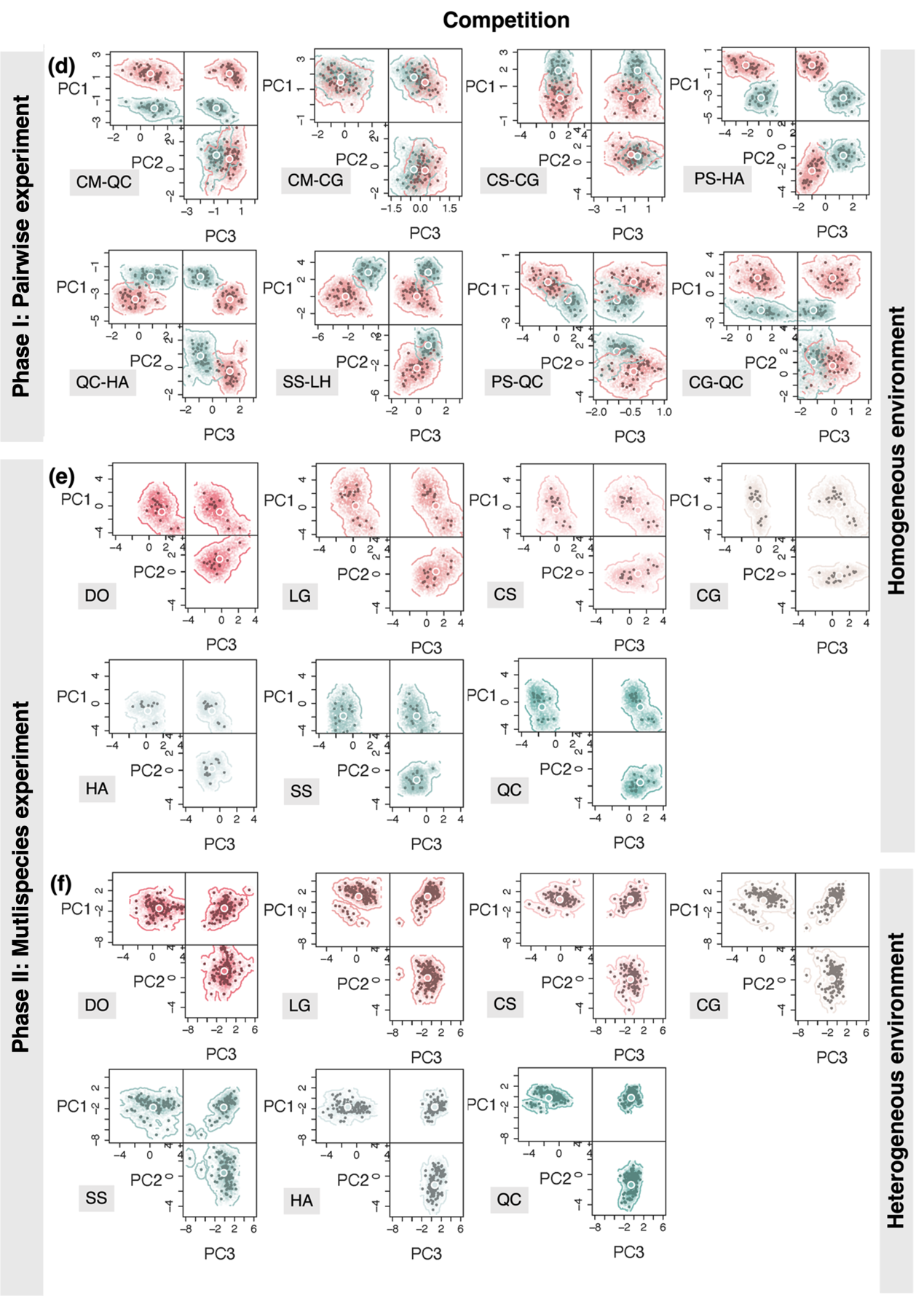


**Fig. S4 |** Hypervolume visualization for competitively inferior species and superior species in other 7 species pairs in a 2-species pairwise competition experiment in a homogeneous environment (a-b), per species in multispecies experiment in homogeneous (c-d, environmental block 1) and heterogeneous (e-f) abiotic environments. The hypervolume was constructed based on the 7 traits considered and presented by the combinations of the first 3 principal components (contained 89.36%, 74.60% and 72.87% of the total trait variation in order). Large points with white borders depict hypervolume centroids and curves describe the hypervolume boundaries to visualize the stochastic description of each hypervolume. Detailed species Latin names for each species pair code are given in Table 1.


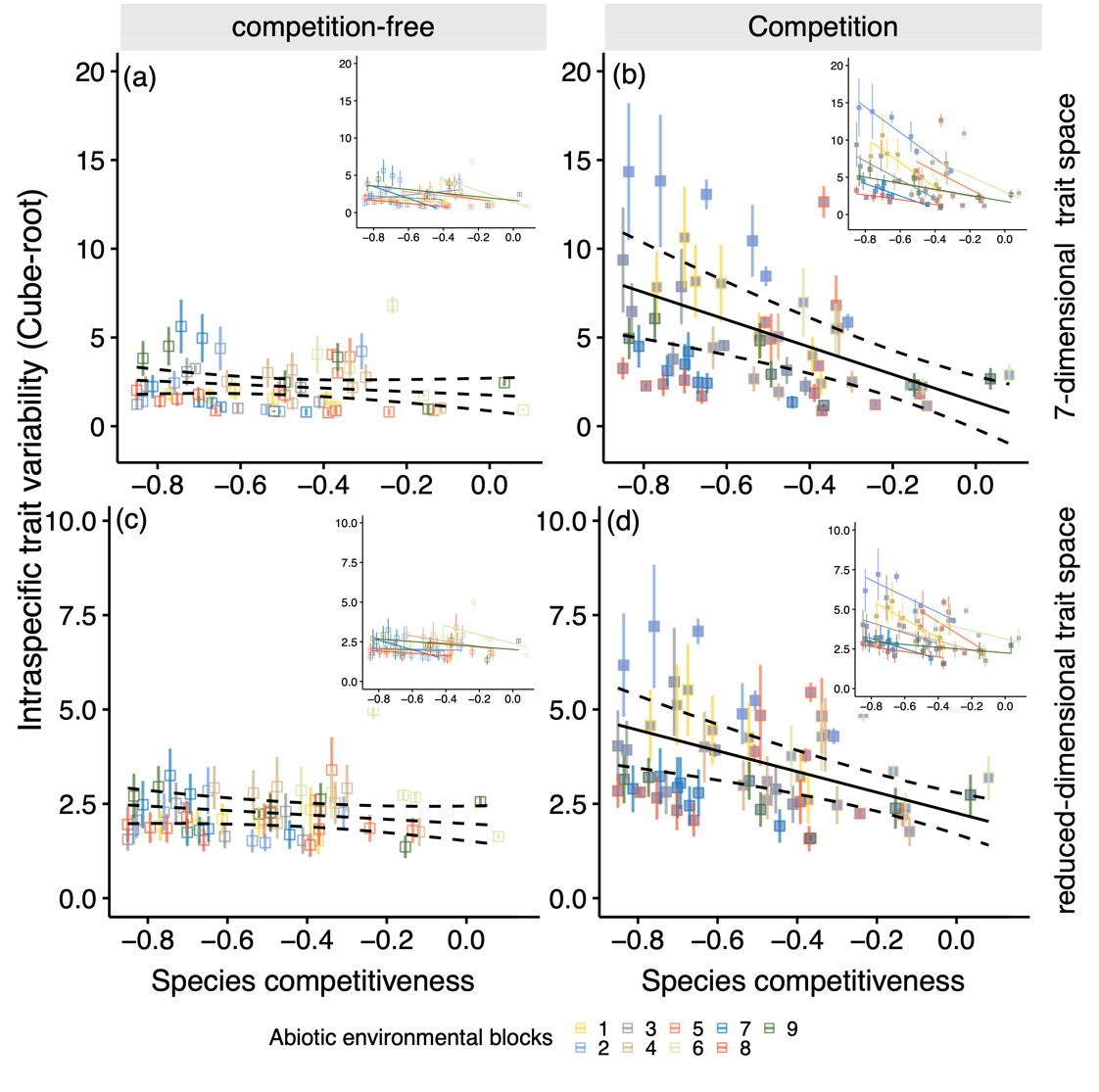


**Fig. S5 |** Overall relationships between intraspecific trait variability (mean ± standard error, cube-root transformation) and competitiveness of species (points) under competition-free (colored hollow points, a, c) and multispecies competition (colored solid points, b, d) in 9 homogeneous environments. The top right panel shows the relationship between intraspecific trait variability and competitiveness within each abiotic environment. Intraspecific trait variability was quantified by hypervolume of the raw 7 traits (a-b) and the reduced dimension first 3 principal axis traits (PC1-PC3, c-d), respectively. Statistically significant relationships (*P* < 0.05), depicted by black solid lines, and non-significant relationships (*P* > 0.05), indicated by black dashed lines, were inferred using mixed-effects models, each encompassing a 95% confidence interval. Similarly, colored solid and dashed lines represent significant and non-significant simple linear regressions, respectively, also with a 95% confidence interval.


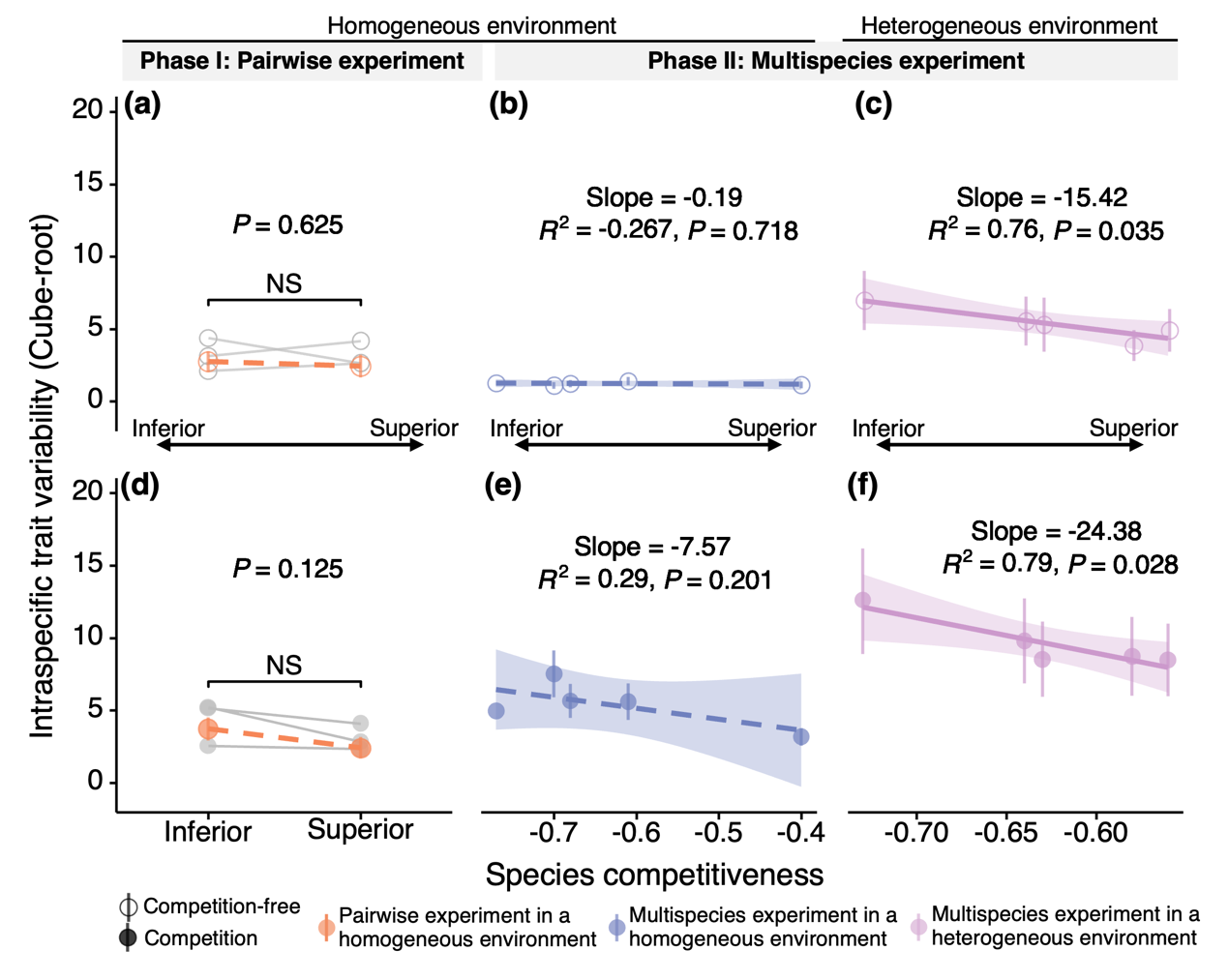


**Fig. S6** | Relationships between intraspecific trait variability (ITV) and competitiveness of five evergreen species. a-c, marked with hollow points, denote competition-free treatments, whereas d-f, marked with solid points, represent three varied competition treatments. Specifically, a, d depicts pairwise experiments in homogeneous environments, b, e and c, f depicts multispecies experiments in homogeneous and heterogeneous environments, respectively. ITV per species was quantified by the 999 simulated hypervolume of seven traits, measured in SD^7^ units. Competitiveness was quantified using the relative interaction intensity index (*RII*) based on whole seedlings’ biomass. Gray (a, d), blue (b, e), and purple (c, f) points with error bars represent the mean and standard error of the 999 simulated hypervolumes for each competing species, transformed by cube root. Detailed sample size for each competing species in two-phase experiment are shown in Table S4. The orange dots in a and d represent the mean ITVs of the eight superior (or inferior) species. The gray and orange line connecting these dots visually depicts the difference in ITV between competing species, and significance was tested by one-sided paired Wilcoxon rank-sum test. Black asterisks indicate levels of significance (•: <0.1; *: <0.05; **: <0.01; ***: <0.001). NS indicates nonsignificant. In b, c and d–f, solid (*P* < 0.05) and dashed (*P* > 0.05) lines are simple linear regression lines with 95% confidence intervals.


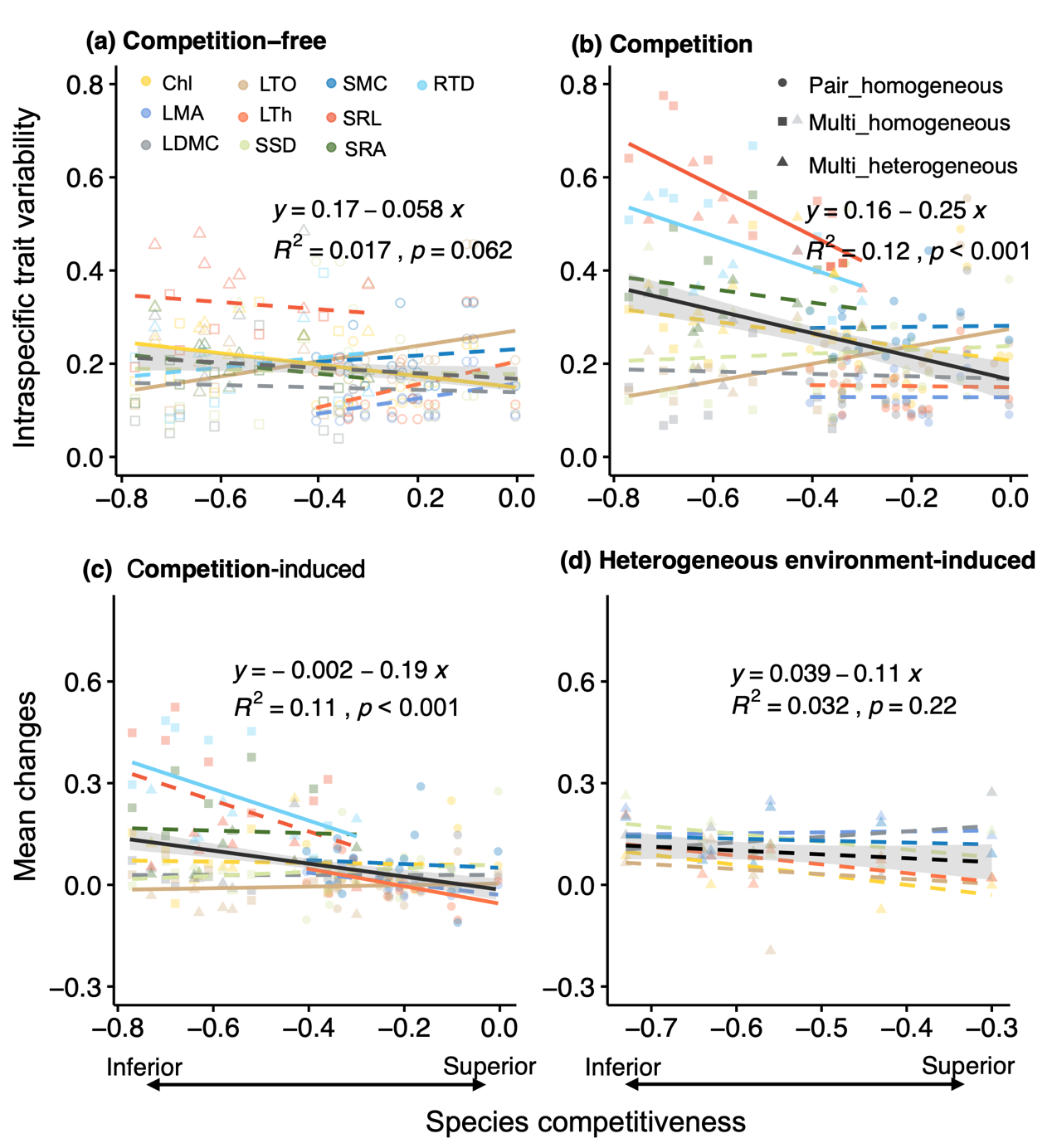


**Fig. S7 |** The relationships between species competitiveness (*RII*) and intraspecific trait variability (ITV) for individual traits of competing species in competition-free (a, hollow points) and competition (b, solid points) treatments under 2-species (round) competition experiments in the homogeneous environments (Phase I), multispecies competition experiments in the homogeneous (square points) and heterogeneous (triangle points) environments (Phase II). Relationship between changes in ITV induced by competition and heterogeneous environments and species competitiveness. Each color denotes a unique trait, and the three point shapes represent the three competition scenarios. The black solid (*P* < 0.05) and dashed (*P* > 0.05) lines are the overall simple linear regression line, and the gray area around the black line is the 95% confidence interval for the regression line. The colored solid (*P* < 0.05) and dashed (*P* > 0.05) lines are simple linear regression lines for individual traits.


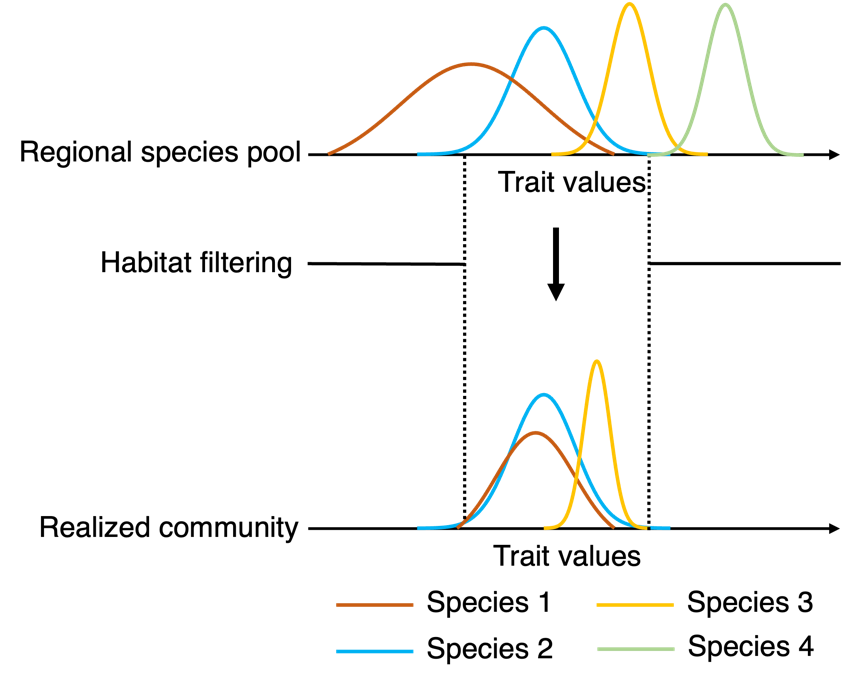


**Fig. S8 |** A conceptual diagram delineates abiotic environmental filtering operating at the individual level across various species (colored trait distribution curves). If all individuals of a species are maladapted, the species go extinction (species 4 with green trait distribution curve). Alternatively, with variable ITV, if a subset of individuals are maladapted, the filter eliminates only these individuals, enabling the persistence of adapted subset (species 1 with red curve). The resulting truncated population passing through this bottleneck exhibits reduced ITV and a changed mean trait value. Essentially, environmental filtering alters trait distribution via this ITV reduction and mean shift.


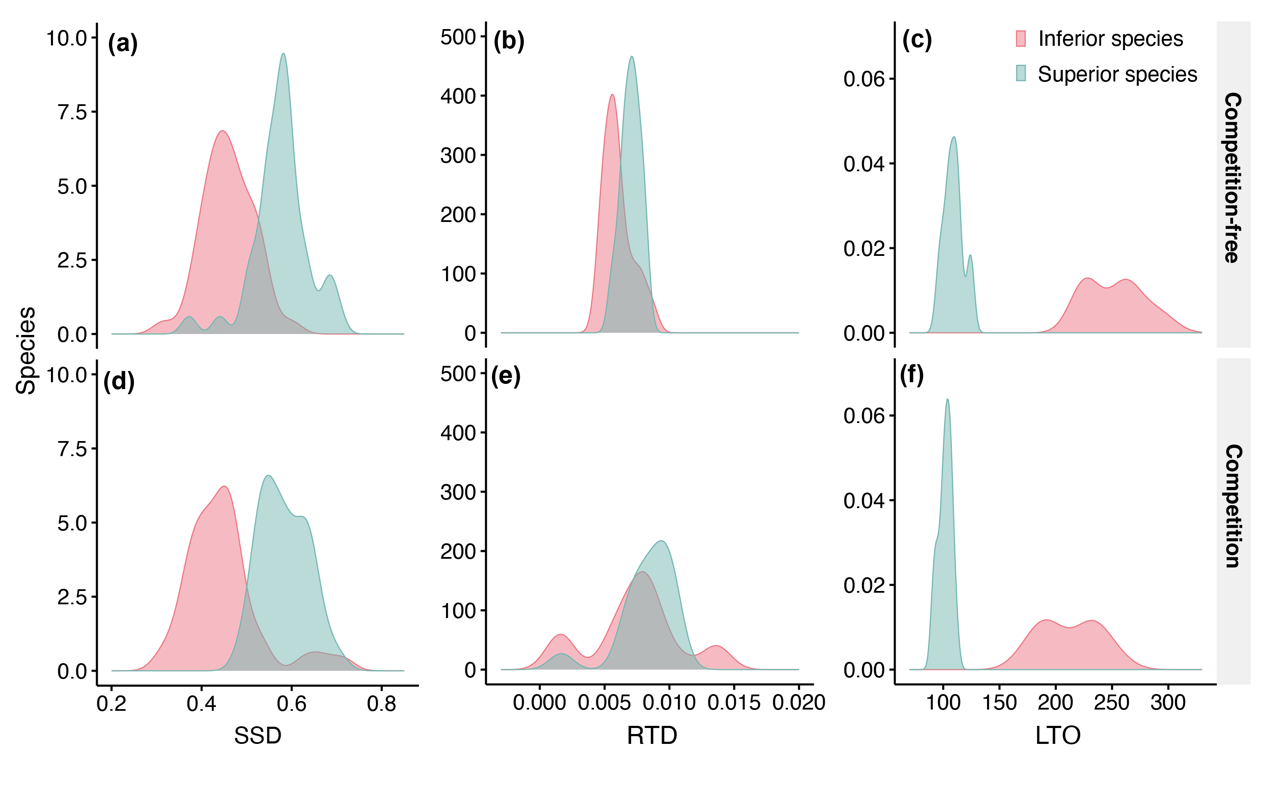


**Fig. S9 |** Examples of trait distributions for competitively inferior and superior species in the competition-free (a-c) and competition (d-f) treatments. Specifically, a and d are the distributions of stem-specific density (SSD) for *Schima superba* (inferior) and *Lithocarpus harlandii* (superior) in two-species competition homogeneous environments, b and e are the distributions of root tissue densities (RTD) for *Daphniphyllum oldhami* (inferior) and *Quercus chenii* (superior) in multispecies competition homogeneous (block 8) environments, c and f are the leaf toughness (LTO) for *Daphniphyllum oldhami* (inferior) and *Quercus chenii* (superior) in multispecies competition homogeneous (block 1) environments.

**
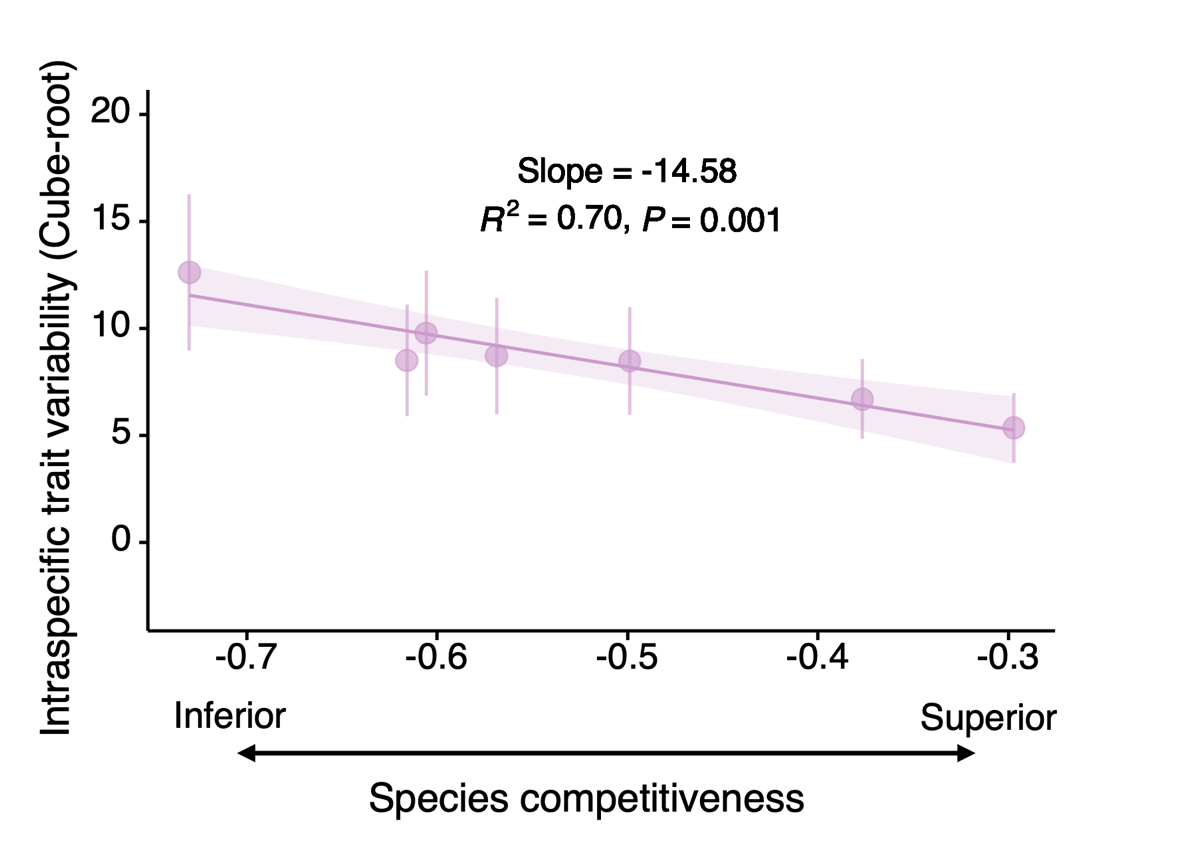
**

**Fig. S10 |** Relationships between changes in intraspecific trait variability (mean ± standard error, cube-root transformation) from competition-free in a homogeneous environment (Figure 2b, environmental block 1) to competition in a heterogeneous environment (Figure 2f). Intraspecific trait variability was quantified by the hypervolume of seven traits, measured in SD^7^ units. Solid (*P* < 0.05) lines are simple linear regression lines with 95% confidence interval.


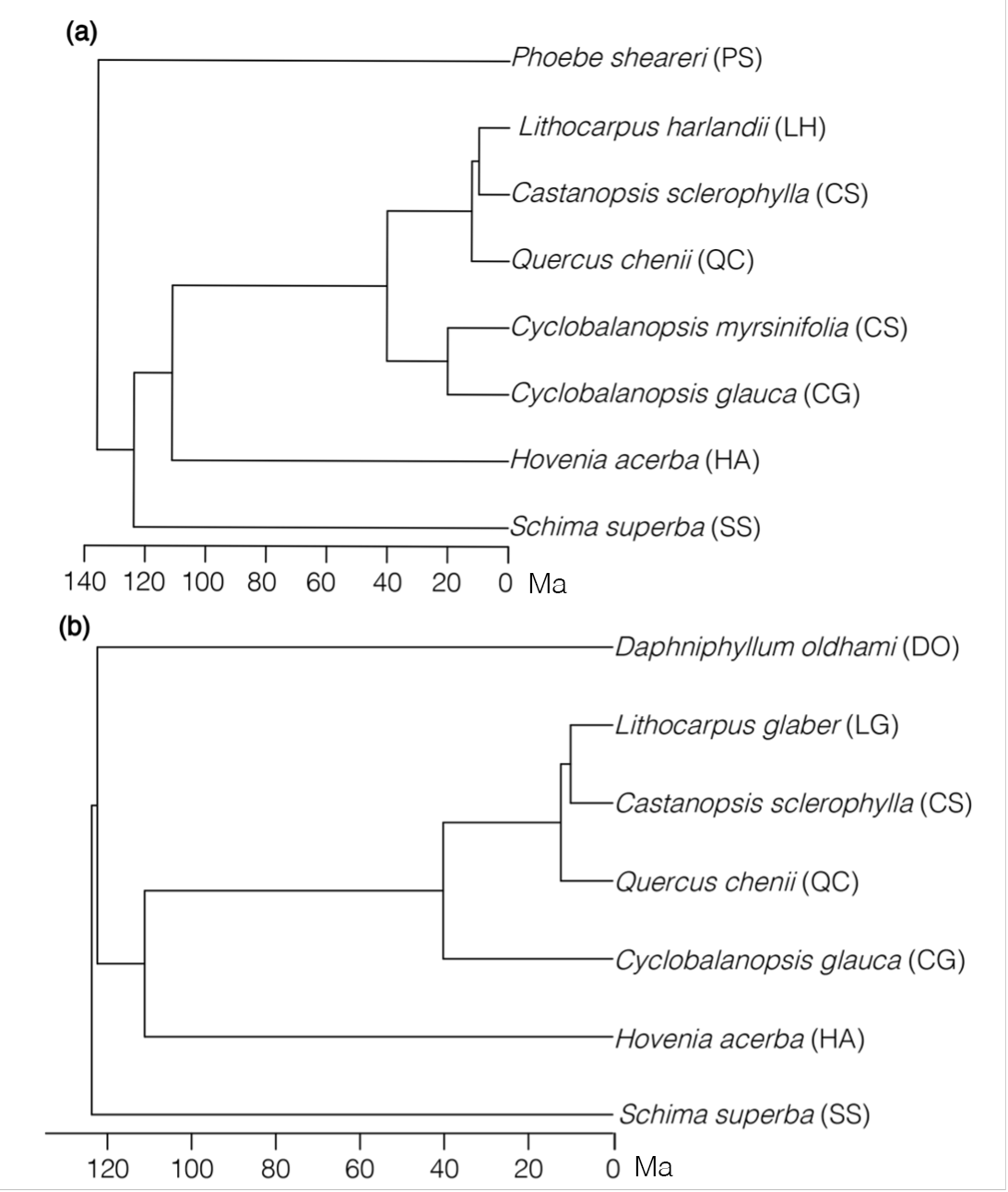


**Fig. S11 |** Phylogenetic trees of the experimental species in Phase I (a) and Phase II (b). This tree was constructed by a mega-tree approach in the R package “V.PhyloMaker”, which is the largest dated phylogeny for vascular plant species.


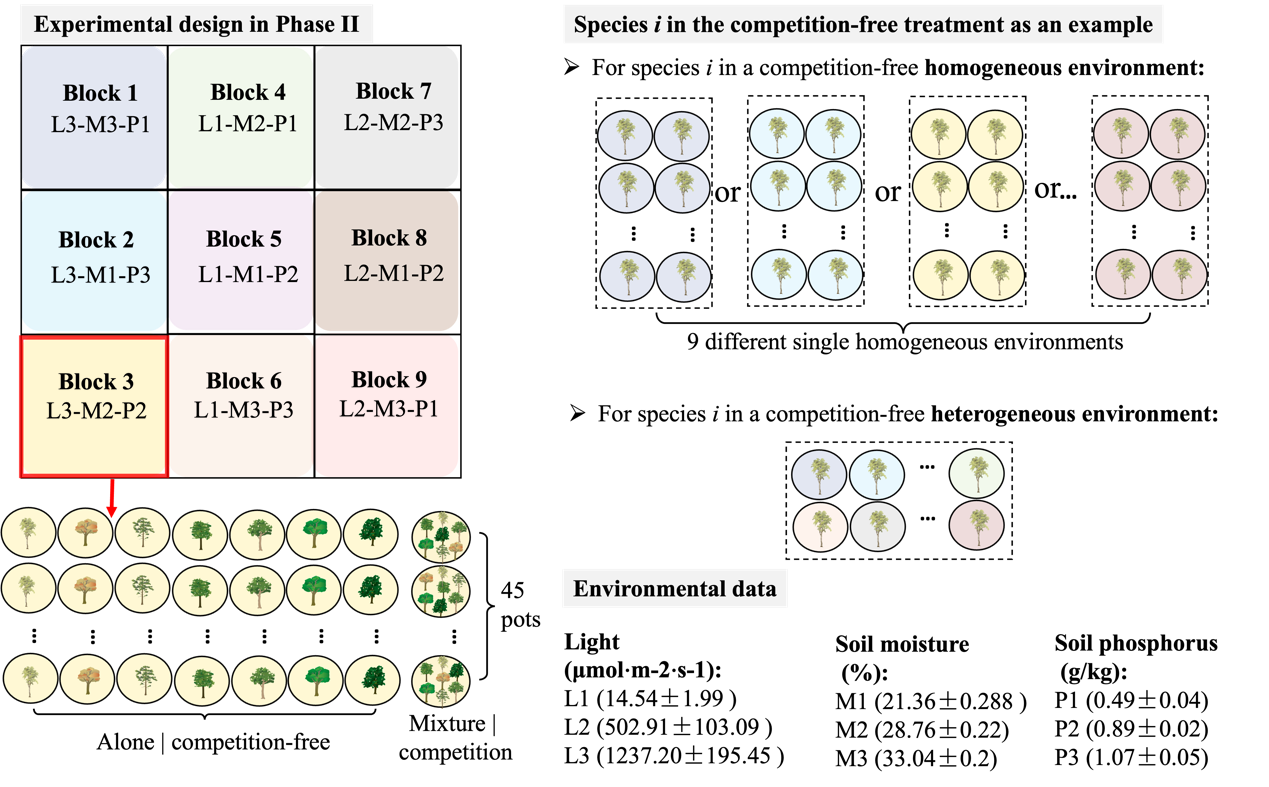


**Fig. S12 |** Detailed schematic diagrams on abiotic environmental conditions, competitive treatments in phase II experiment, and species definitions in both homogeneous and heterogeneous environments. First, nine different homogeneous abiotic environments (labeled Block 1 to Block 9) were set up based on a three-factor, three-level orthogonal experimental design. The three abiotic factors were light intensity, soil moisture, and soil phosphorus content. The environmental data for the three levels (L1-L3, M1-M3, and P1-P3) are presented in the bottom right corner (mean ± standard error). Each environment included both alone planting and mixed planting. Different background colors represent different environmental blocks. Taking the example of species *i* under competition-free treatment, the seedlings of species *i* from a single abiotic environment represented the population of species *i* in the competition-free homogeneous environment, resulting in a total of nine different homogeneous environments. Furthermore, the seedlings of species *i* from the multiple different abiotic environments together constituted the population of species *i* in the competition-free heterogeneous environment. Using this setup, we quantified the intraspecific trait variability and competitiveness of species under competition-free treatments in both homogeneous and heterogeneous environments. The visual elements used in this figure are provided by the Integration and Application Network (IAN, ian.umces.edu) at the University of Maryland Center for Environmental Science (UMCES), which are available under the Creative Commons Attribution-ShareAlike 4.0 International (CC BY-SA 4.0) license (https://creativecommons.org/licenses/by-sa/4.0/).

**
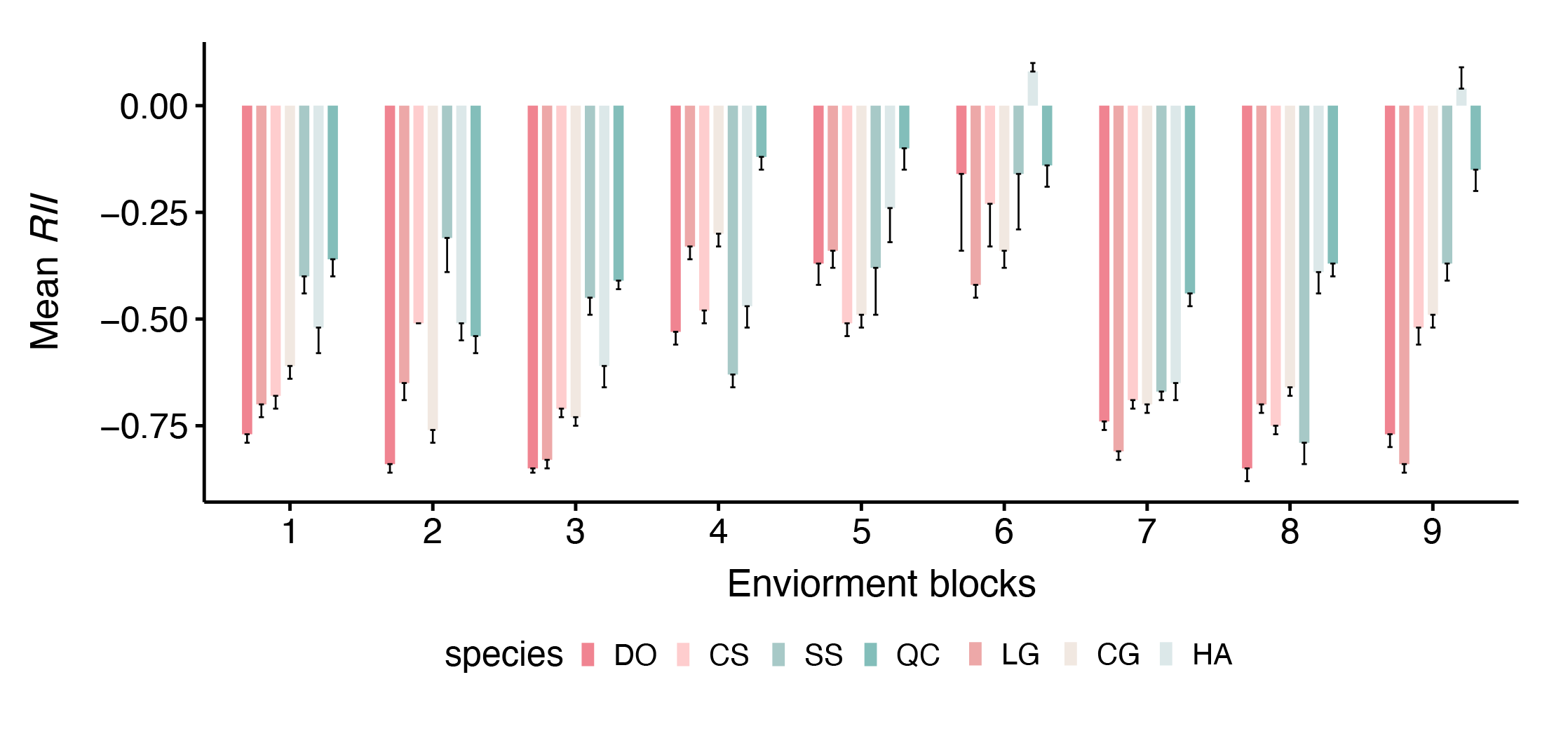
 Fig. S13 |** Competition intensity (*RII*) per species within nine different single environmental blocks in multispecies competition experiments (Phase II).


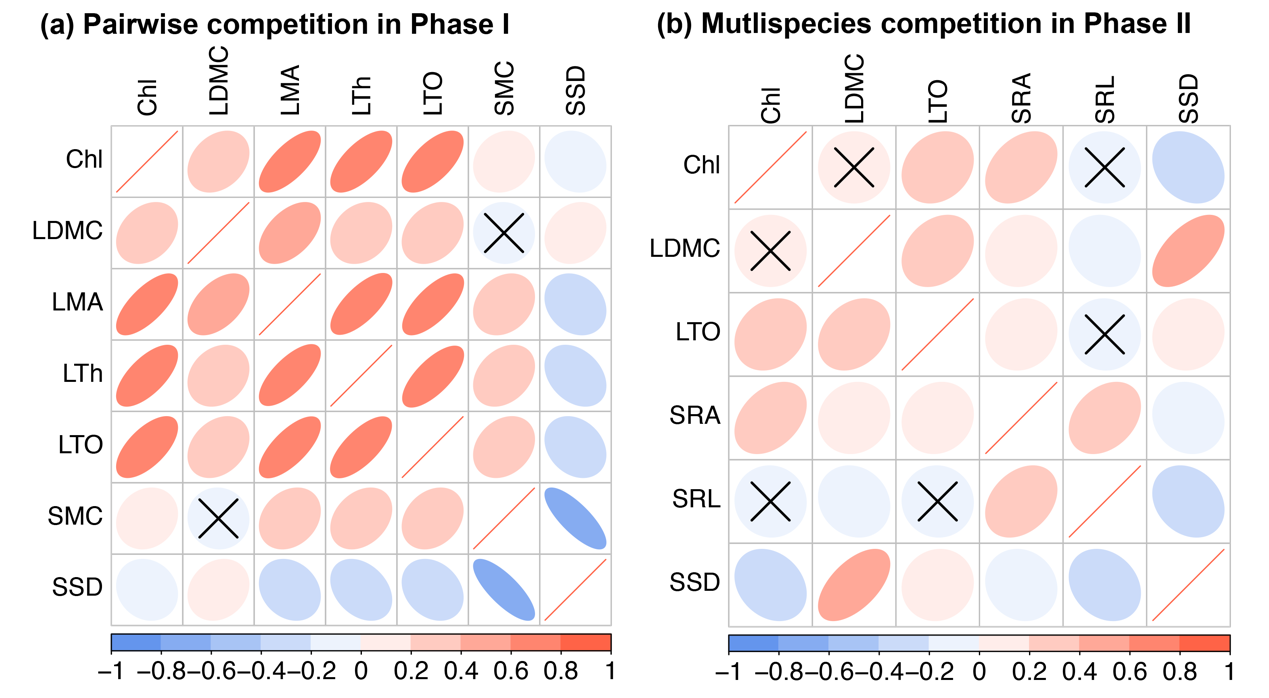


**Fig. S14 |** Pearson correlation plot for functional traits in 2-species pairwise competition in Phase I (a), multispecies competition experiments in Phase II (b). The direction of correlation is indicated by color type (red: positive; blue: negative). The strength of correlation is indicated by color intensity; uncertainty around the estimates is indicated by width of the ovals (wider: more uncertainty). Only correlations statistically significant at *P* < 0.05 unless crossed with an X.


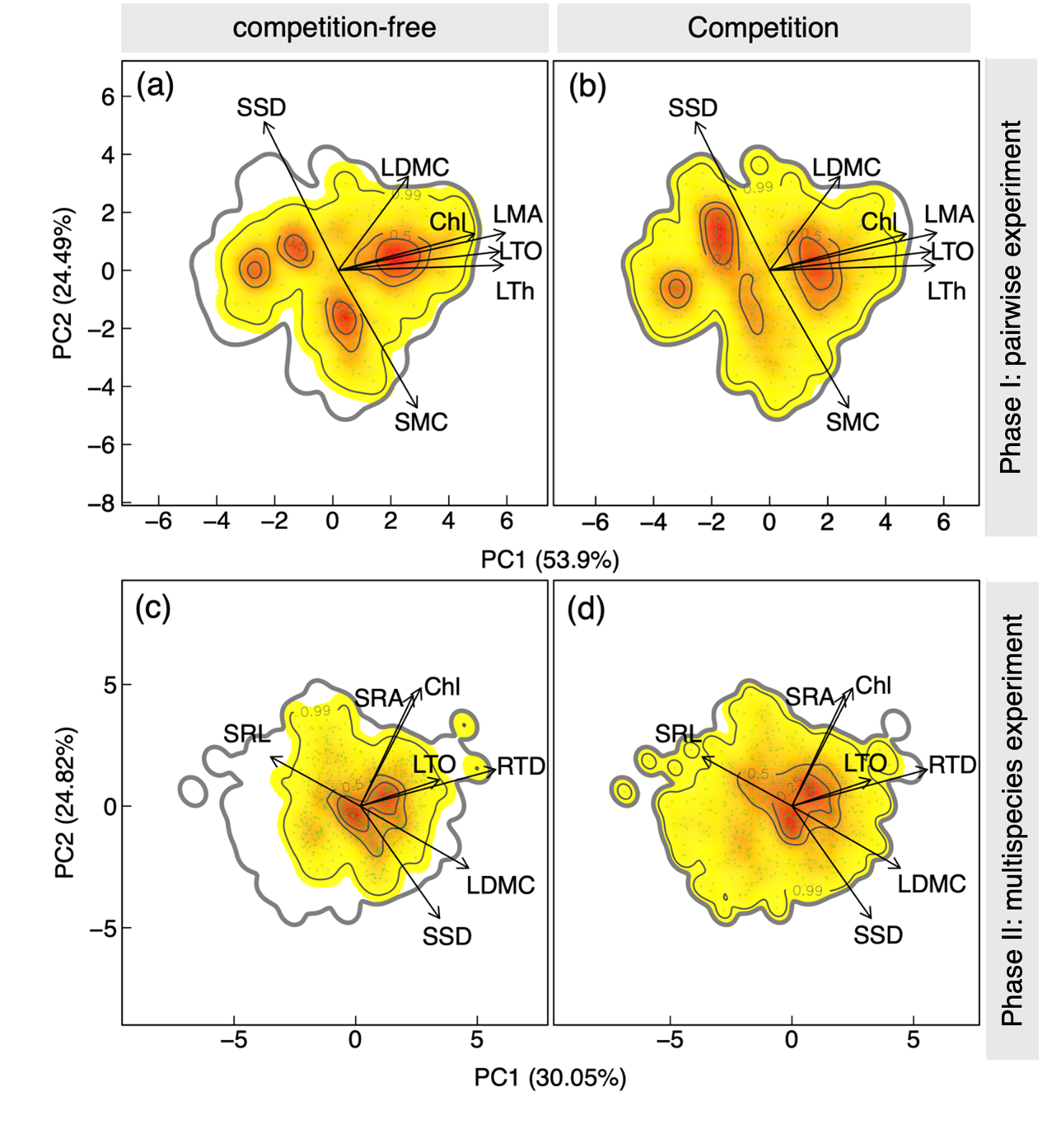
**Fig. S15 |** Probability species distributions in space based on PCA-measured functional traits in competition-free (left panels) and competition (right panels) treatments for Phase I (a, b) and Phase II (c, d) experiments. Colors indicate the probability distribution of trait combinations in the space of functional traits defined by PCA (the red area is high probability, and yellow area is low probability). Contours indicate the 0.99, 0.50, and 0.25 magnitudes of the probability distribution. The functional trait space was calculated and plotted by the “funspace” R package. Detailed functional trait information is shown in Table S9.
