# Supplementary material for: Inverse relationship between species competitiveness and intraspecific trait variability may enable species coexistence in experimental seedling communities
Source: Nat Commun. 2024 Apr 3;15:2895. doi: 10.1038/s41467-024-47295-4 (PMC10991546; doi:10.1038/s41467-024-47295-4)
Supplement: Supplementary file 3 — Reporting Summary [file 41467_2024_47295_MOESM3_ESM.pdf]

nature portfolio

Corresponding author(s): Guochun Shen

Last updated by author(s): Mar 10, 2024

# Reporting Summary

Nature Portfolio wishes to improve the reproducibility of the work that we publish. This form provides structure for consistency and transparency in reporting. For further information on Nature Portfolio policies, see our Editorial Policies and the Editorial Policy Checklist.

## Statistics

For all statistical analyses, confirm that the following items are present in the figure legend, table legend, main text, or Methods section.

| n/a | Confirmed | |
|---|---|---|
| ☐ | ☒ | The exact sample size (*n*) for each experimental group/condition, given as a discrete number and unit of measurement |
| ☐ | ☒ | A statement on whether measurements were taken from distinct samples or whether the same sample was measured repeatedly |
| ☐ | ☒ | The statistical test(s) used AND whether they are one- or two-sided<br>*Only common tests should be described solely by name; describe more complex techniques in the Methods section.* |
| ☐ | ☒ | A description of all covariates tested |
| ☐ | ☒ | A description of any assumptions or corrections, such as tests of normality and adjustment for multiple comparisons |
| ☐ | ☒ | A full description of the statistical parameters including central tendency (e.g. means) or other basic estimates (e.g. regression coefficient) AND variation (e.g. standard deviation) or associated estimates of uncertainty (e.g. confidence intervals) |
| ☐ | ☒ | For null hypothesis testing, the test statistic (e.g. *F*, *t*, *r*) with confidence intervals, effect sizes, degrees of freedom and *P* value noted<br>*Give P values as exact values whenever suitable.* |
| ☒ | ☐ | For Bayesian analysis, information on the choice of priors and Markov chain Monte Carlo settings |
| ☐ | ☒ | For hierarchical and complex designs, identification of the appropriate level for tests and full reporting of outcomes |
| ☐ | ☒ | Estimates of effect sizes (e.g. Cohen's *d*, Pearson's *r*), indicating how they were calculated |

*Our web collection on statistics for biologists contains articles on many of the points above.*

## Software and code

Policy information about availability of computer code

| | |
|---|---|
| Data collection | No software was used for data collection. |
| Data analysis | All analyses were conducted using R software (version 4.0.5). We used the "hypervolume" R package (v.3.1.1) to calculate and visualize intraspecific trait variability based on multidimensional traits. We used the "funspace" R package (v.0.1.1) to visualize the functional trait space of the species. We utilized the "parallel" R package (v.4.3.0) to enhance computational efficiency of hypervolume through parallel operations. We performed linear mixed-effects models and a binomial generalized linear mixed model using the "lme4" R package (v.1.1.33). Standardized effect sizes of variables were calculated using the R package "effectsize" (v.0.8.3). Additionally, figures were generated using the "ggplot2" R package (v.3.4.4). All R scripts used for statistical analyses and plotting are available on the Figshare digital repository (https://doi.org/10.6084/m9.figshare.24174558.v3), GitHub (https://github.com/Jingyangecnu/Inverse-relationship-between-species-competitiveness-and-intraspecific-trait-variability), and Zenodo (https://zenodo.org/records/10794876). |

For manuscripts utilizing custom algorithms or software that are central to the research but not yet described in published literature, software must be made available to editors and reviewers. We strongly encourage code deposition in a community repository (e.g. GitHub). See the Nature Portfolio guidelines for submitting code & software for further information.

## Data

Policy information about availability of data

All manuscripts must include a data availability statement. This statement should provide the following information, where applicable:

- Accession codes, unique identifiers, or web links for publicly available datasets
- A description of any restrictions on data availability
- For clinical datasets or third party data, please ensure that the statement adheres to our policy

The data that support the findings of this study are available on the Figshare digital repository (https://doi.org/10.6084/m9.figshare.24174558.v3), GitHub (GitHub - Jingyangecnu/Inverse-relationship-between-species-competitiveness-and-intraspecific-trait-v), and Zenodo (https://zenodo.org/records/10794876).

## Research involving human participants, their data, or biological material

Policy information about studies with human participants or human data. See also policy information about sex, gender (identity/presentation), and sexual orientation and race, ethnicity and racism.

| | |
|---|---|
| Reporting on sex and gender | NA |
| Reporting on race, ethnicity, or other socially relevant groupings | NA |
| Population characteristics | NA |
| Recruitment | NA |
| Ethics oversight | NA |

Note that full information on the approval of the study protocol must also be provided in the manuscript.

# Field-specific reporting

Please select the one below that is the best fit for your research. If you are not sure, read the appropriate sections before making your selection.

☐ Life sciences ☐ Behavioural & social sciences ☒ Ecological, evolutionary & environmental sciences

For a reference copy of the document with all sections, see nature.com/documents/nr-reporting-summary-flat.pdf

# Ecological, evolutionary & environmental sciences study design

All studies must disclose on these points even when the disclosure is negative.

| | |
|---|---|
| Study description | To investigate the inverse relationship between species' competitive ability and intraspecific trait variability (ITV) across various competition scenarios, we conducted a two-phase seedling competition experiment spanning three years. In Phase I (2017-2018), we selected eight subtropical evergreen tree species and eight species pairs based on life type and phylogenetic relationships. We then established two planting scenarios: alone and paired, with 45 pot replicates for each species or pair. These setups allowed us to quantify ITV and species' competitiveness, enabling us to explore the existence of a negative relationship. In phase II (2019-2021), we expanded our study to incorporate multi-species competition across homogeneous and heterogeneous abiotic environments. We focused on seven subtropical evergreen tree species and established nine distinct abiotic environments by manipulating gradients of light intensity, soil moisture, and soil phosphorus content, following the principles of orthogonal experimental design. Each abiotic environment included both alone and mixed planting setups, with 45 pot replicates for each species under each planting. These configurations allowed us to quality the species competitiveness and ITV, and test whether the negative relationship persisted in more complex competitive and abiotic conditions. |
| Research sample | Initially, we selected 16 tree species for seed collection and subsequent seedling development based on their growth form (11 evergreen and 5 deciduous species), seed availability, and phylogenetic relationships. However, due to lower than expected germination rates for some of the species, we ultimately reduced the number of experimental species to 10, with 8 species in Phase I and 7 species in Phase II. |
| Sampling strategy | The Phase I experiment involved 8 species, with 45 replicates for each species in the competition-free treatment, and 45 replicates for each species pair in the competitive treatment, resulting in a total of 720 pots and 1080 seedlings. In the Phase II experiment, we included 7 species and 9 different abiotic environmental block groups. For each species, we conducted 45 pot replicates in the competition-free treatment and 45 pot replicates in the competitive treatment under each environmental block group, resulting in a total of 3240 pots and 5670 seedlings. It should be noted that the determination of sample size is based on the results of Yang et al.'s 2021 (DOI: 10.3389/fpls.2020.00053), which found that small sample (sizes such as 5) can lead to a significant underestimation of ITV. To accurately quantify the ITV of species, an adequate sample size of 20 or more should be used, along with an improved |

| | |
|---|---|
| | coefficient of variation (Bao's CV). Therefore, this study chose a sample size of 45 to ensure that there are still more than 20 samples available for accurate estimation of ITV even when the survival rate of seedlings is high. |
| Data collection | Data for this study were collected by Jing Yang, Xiya Wang, Qiuyu Yu, Siyu Wu, Congling Zhang, Ruijiao Jiang, Yue Chen and Jiahui Lu, with assistance from trained assistants. The growth and mortality of each seedling were monitored during two-phase experiment conducted from 2017-2018 and 2019-2021, respectively. After one year of experimentation in Phase I (and 2 years in Phase I), we measured 10 key functional traits, as well as above- and below-ground biomass of each seedling, following a standardized protocol. These traits included leaf relative chlorophyll content, leaf dry matter content, leaf mass, leaf toughness, leaf thickness, stem specific density, stem water content, root specific length, root specific area, and root tissue density. |
| Timing and spatial scale | We collected more than 2,000 intact seeds of each species in 2016 and 2018 for Phase I and Phase II, respectively. After collection, we sterilized the seeds with insecticides, stored them throughout the winter, and sowed them in seedling trays in April of the corresponding experimental years. In June, we selected individuals with similar growth rates for transplantation and established the experimental treatments. Throughout the experiment, we monitored the growth and mortality of each seedling quarterly. After one year of the experiment (2 years in Phase II), we measured 10 key functional traits, above- and below-ground biomass of the seedlings using a standardized protocol. |
| Data exclusions | When estimating intraspecific trait variability, we removed dead and missing trait values of seedlings in Phase I and Phase II. |
| Reproducibility | This study conducted sequential competition experiments ranging from two-species competitive systems to multi-species competitive systems, and from homogeneous to different abiotic environments. These variations in experimental conditions increase our confidence in the generalizability of the observed effects. |
| Randomization | The study included species that were chosen at random from a pool of candidates that met the experimental criteria. Furthermore, the placement of pots in the greenhouse trellis and the assignment of sampling tasks to field assistants were both randomized. |
| Blinding | Experimental treatments were randomized within each pot, and the spatial positioning of the pots within each environmental block was randomized approximately once a week. All of the pots used in the experiment were identical in shape, size, and color. These procedures were put in place to ensure that the researchers were not biased in their observations of the experimental treatments. |

Did the study involve field work? ☐ Yes ☒ No

# Reporting for specific materials, systems and methods

We require information from authors about some types of materials, experimental systems and methods used in many studies. Here, indicate whether each material, system or method listed is relevant to your study. If you are not sure if a list item applies to your research, read the appropriate section before selecting a response.

## Materials & experimental systems

| n/a | Involved in the study | |
|---|---|---|
| ☒ | ☐ | Antibodies |
| ☒ | ☐ | Eukaryotic cell lines |
| ☒ | ☐ | Palaeontology and archaeology |
| ☒ | ☐ | Animals and other organisms |
| ☒ | ☐ | Clinical data |
| ☒ | ☐ | Dual use research of concern |
| ☐ | ☒ | Plants |

## Methods

| n/a | Involved in the study | |
|---|---|---|
| ☒ | ☐ | ChIP-seq |
| ☒ | ☐ | Flow cytometry |
| ☒ | ☐ | MRI-based neuroimaging |

# Dual use research of concern

Policy information about dual use research of concern

## Hazards

Could the accidental, deliberate or reckless misuse of agents or technologies generated in the work, or the application of information presented in the manuscript, pose a threat to:

| No | Yes | |
|---|---|---|
| ☒ | ☐ | Public health |
| ☒ | ☐ | National security |
| ☒ | ☐ | Crops and/or livestock |
| ☒ | ☐ | Ecosystems |
| ☒ | ☐ | Any other significant area |

## Experiments of concern

Does the work involve any of these experiments of concern:

| No | Yes | |
|---|---|---|
| ☒ | ☐ | Demonstrate how to render a vaccine ineffective |
| ☒ | ☐ | Confer resistance to therapeutically useful antibiotics or antiviral agents |
| ☒ | ☐ | Enhance the virulence of a pathogen or render a nonpathogen virulent |
| ☒ | ☐ | Increase transmissibility of a pathogen |
| ☒ | ☐ | Alter the host range of a pathogen |
| ☒ | ☐ | Enable evasion of diagnostic/detection modalities |
| ☒ | ☐ | Enable the weaponization of a biological agent or toxin |
| ☒ | ☐ | Any other potentially harmful combination of experiments and agents |

# Plants

| | |
|---|---|
| Seed stocks | NA |
| Novel plant genotypes | NA |
| Authentication | NA |
